# Stand carbon storage and net primary production in China’s subtropical secondary forests are predicted to increase by 2060

**DOI:** 10.1186/s13021-022-00204-y

**Published:** 2022-05-26

**Authors:** Jia Jin, Wenhua Xiang, Yelin Zeng, Shuai Ouyang, Xiaolu Zhou, Yanting Hu, Zhonghui Zhao, Liang Chen, Pifeng Lei, Xiangwen Deng, Hui Wang, Shirong Liu, Changhui Peng

**Affiliations:** 1 Faculty of Life Science and Technology, Central South University of Forestry and Technology, No. 498 Southern Shaoshan Road, Changsha, 410004 Hunan China; 2 Huitong National Station for Scientific Observation and Research of Chinese Fir Plantation Ecosystems in Hunan Province, Huitong, 438107 Hunan China; 3 School of Geographic Sciences, Hunan Normal University, Changsha, 410081 China; 4 Research Institute of Forest Ecology, Environment and Protection, Chinese Academy of Forestry, Beijing, 100091 China; 5 Department of Biological Sciences, Institute of Environment Sciences, University of Quebec at Montreal, Montreal, QC H3C 3P8 Canada

**Keywords:** Carbon (C) storage, Climate change, Forest restoration, Net primary production (NPP), TRIPLEX model

## Abstract

**Background:**

Forest ecosystems play an important role in carbon sequestration, climate change mitigation, and achieving China's target to become carbon (C) neutral by 2060. However, changes in C storage and net primary production (NPP) in natural secondary forests stemming from tree growth and future climate change have not yet been investigated in subtropical areas in China. Here, we used data from 290 inventory plots in four secondary forests [evergreen broad-leaved forest (EBF), deciduous and evergreen broad-leaved mixed forest (DEF), deciduous broad-leaved forest (DBF), and coniferous and broad-leaved mixed forest (CDF)] at different restoration stages and run a hybrid model (TRIPLEX 1.6) to predict changes in stand carbon storage and NPP under two future climate change scenarios (RCP4.5 and RCP8.5).

**Results:**

The runs of the hybrid model calibrated and validated by using the data from the inventory plots suggest significant increase in the carbon storage by 2060 under the current climate conditions, and even higher increase under the RCP4.5 and RCP8.5 climate change scenarios. In contrast to the carbon storage, the simulated EBF and DEF NPP declines slightly over the period from 2014 to 2060.

**Conclusions:**

The obtained results lead to conclusion that proper management of China’s subtropical secondary forests could be considered as one of the steps towards achieving China’s target to become carbon neutral by 2060.

**Supplementary Information:**

The online version contains supplementary material available at 10.1186/s13021-022-00204-y.

## Background

Forests cover 4.03 billion hectares worldwide, which is about 30% of the terrestrial surface of our planet, and account for 45% of terrestrial C and 75% of terrestrial gross primary production (GPP) [1, 2]. Forests play an important role in the global C cycle [3, 4]. Vegetation NPP measures the direct production capacity of forest ecosystems and C sequestration efficiency in the terrestrial C cycle [5, 6]. Attaining net zero emissions by mid-century will require all nations to comply with the objectives and principles laid out in the UN Framework Convention on Climate Change (UNFCCC) and its Paris Agreement. Accurate estimation of forest C storage and potential capacity is essential for achieving this goal. The current rate of change in global ecosystems is unprecedented. Forests can sequester C in ecosystems [3], and this is a safe and affordable strategy for mitigating the effects of climate change [7]. Recently, the Chinese government officially announced its goal to peak CO_2_ emissions before 2030 and achieve C neutrality in 2060. There is thus an urgent need to determine how the NPP and C sequestration capability of forests might change in the future (especially in 2030 and 2060) under climate change.

Natural forest restoration is an effective approach for storing C [7, 8]. On average, natural forests have 6 times and 40 times C storage capacity of agroforests and plantations, respectively (sequestering 12, 1.9, and 0.3 Pg C per 100 M ha by 2100, respectively) [7]. Therefore, natural forest restoration on disturbed lands should be prioritized [7]. The most effective places for natural forest restoration for C storage are in the tropics and subtropics. Subtropical forests cover 11% of the land surface and store significant amounts of C [9]. There are also large areas of naturally restored young forest on bare land in mountainous areas [10]. Some EBF (the climax vegetation) have been converted into secondary forests via anthropogenic disturbance [7, 10]. Thus, subtropical secondary forests have become one of the most common types of natural forests that have a complex floristic composition and structure [11]. The C sequestration ability of plantations and some natural forests has been well characterized, but our understanding of the role of secondary forests in sequestering C and mitigating climate change is limited [12].

Previous studies have shown that the C sequestration ability of forest ecosystems is affected by the interaction of various factors, such as forest origin, forest type, stand age, geography, and soil conditions [13, 14]. The C sequestration capacity varies greatly among forest types [3]. C storage and NPP were significantly higher in broad-leaved forest than in CDF in a subtropical area [15]. Stand age affects forest C storage and is an important variable for predicting future C sequestration [14, 16]. The growth of forests typically changes with forest age, and C stocks tend to be higher in old, complex forests [12, 14, 17, 18]. Global climate change will affect the structure and functions of forest ecosystems [19, 20]. According to the IPPC5, surface mean temperature is projected to increase in the subtropical region, and precipitation is projected to decrease (RCPs) [21]. The warming tendency from 2011 to 2100 is 0.06 °C/10 a for RCP2.6, 0.24 °C/10 a for RCP4.5, and 0.63 °C/10 a for RCP8.5 [22]. Precipitation will decrease by about 10% in subtropical China over the next century. Climate factors, such as temperature and precipitation, affect forest productivity, plant physiology, and community composition through the specific climatic constraints associated with different forests [23, 24]. Consequently, there is much uncertainty in the direction and magnitude of these effects on different forests [20, 25, 26]. More work is needed to investigate the ecosystem production and C storage capacity in subtropical secondary forests with different stand age and predict changes under future climate change conditions [26, 27]. The stabilization scenario that assumed the imposition of emissions mitigation policies [28] (RCP4.5) and the high—greenhouse gas concentration scenario that does not include any specific climate mitigation target [29] (RCP8.5) will serve as representatives [30], such work would aid the ability of forest managers to manage forests under future climate change.

The C storage and NPP in forests can be estimated using conversion parameter estimation, remote sensing, and model simulation. The models for simulating C storage and productivity are grouped into empirical statistical models based on inventory data of stand characteristics and site conditions [31, 32], mechanistic models based on the interaction between the physiological process of forest growth and environmental factors [33–35], and hybrid models based on statistical experience, environmental change, and a combination of forest physiological and ecological processes [36]. The use of inventory, satellite, and field data for initialization, parameterization, and validation in process-based hybrid models is a promising approach for regional estimation and prediction in future and climate change [37–39]. TRIPLEX1.6 is a hybrid model [36] with simple inputs and high simulation accuracy [40]. Compared with models that have been developed with a static climate or the classical assumption of stable site conditions, the TRIPLEX1.6 model can project the impact of climate change. Therefore, the TRIPLEX1.6 model is a suitable method for simulating and predicting C storage and NPP in subtropical secondary forest under future climate change.

Hunan Province located the subtropical region of southern China and has various secondary forests [41]. We used national forest inventory plot data across Hunan Province and the TRIPLEX1.6 model to simulate stand C storage and NPP of subtropical secondary forests and predict the effects of two future climate change scenarios (RCP4.5 and RCP8.5) on stand C storage and NPP in different forest types. Specifically, the objectives of this study were to (1) characterize differences in C storage and NPP among forest types; (2) predict stand C storage and NPP in different forest types in 2030 and 2060; and (3) quantifying the impacts of stand age and climate change (RCP4.5 and RCP8.5) on C storage and NPP secondary forests in 2030 and 2060. The results of this study can provide a reliable data in C storage of natural secondary forest to help forest managers achieving the C neutrality target in 2060.

## Results

### TIPLEX1.6 model validation

**Fig. 1:**
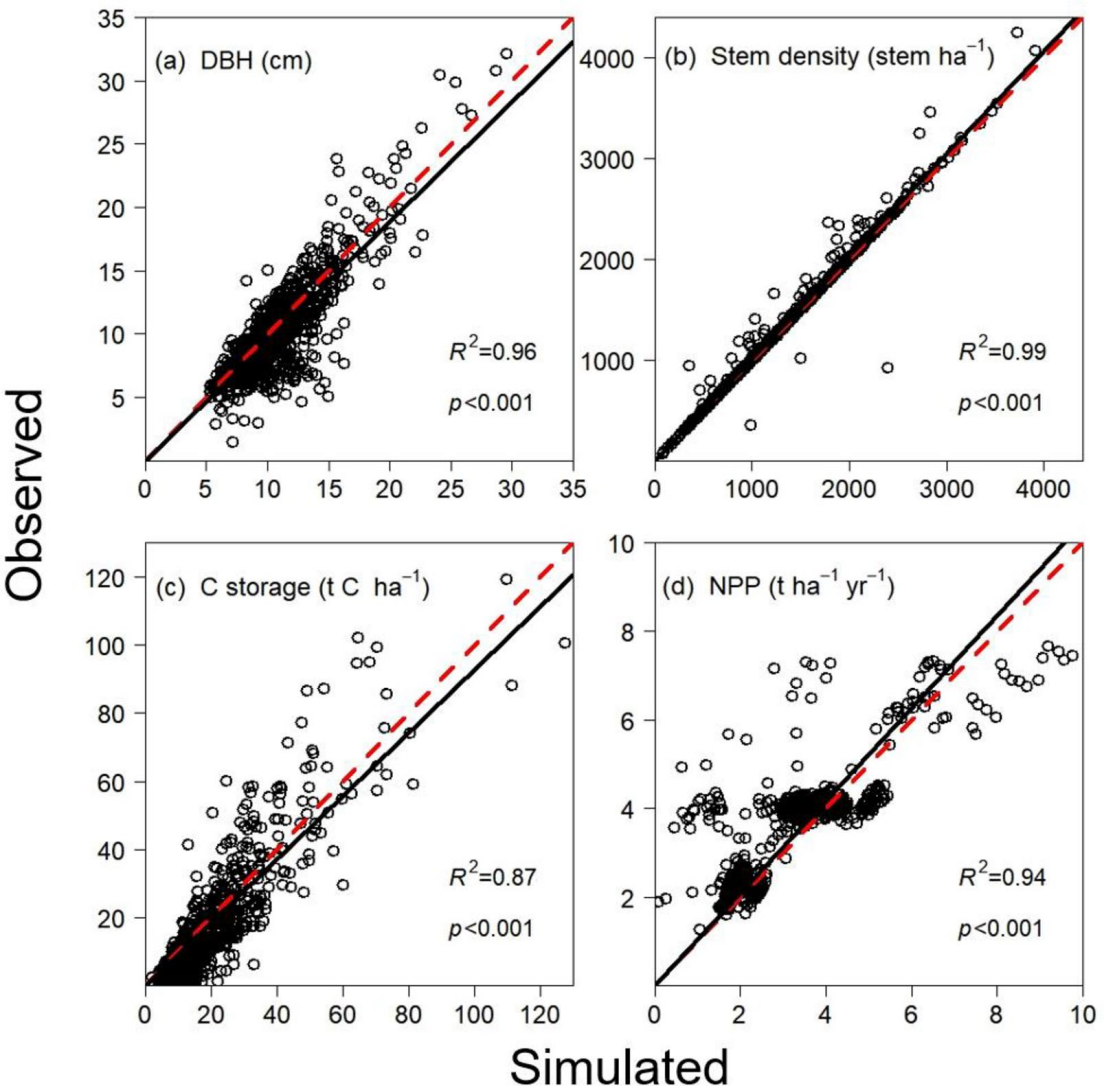
Comparison between the simulated and observed values of **a** DBH (cm); **b** stem density (stem ha^−1^); **c** C storage (t C ha^−1^), and **d** NPP (t ha^−1^ yr^−1^)

We used 290 forest stands according to the proportion of different forest types to calibrate the TRIPLEX1.6 model, and the remainder stands were conducted to validate the prediction of stem density, DBH, C storage and NPP. The simulated values were compared against the observations of all secondary forests to test the prediction accuracy of the model. There were high correlations between the simulated and observed values for stem density (*R*^2^ = 0.99, *p* < 0.01), stand average DBH (*R*^2^ = 0.96, *p* < 0.01), stand C storage (*R*^2^ = 0.87, *p* < 0.01), and NPP (*R*^2^ = 0.94, *p* < 0.01) in all forest stands (Fig. 1).

### Dynamics of stand C storage and NPP with stand age among secondary forests

**Fig. 2:**
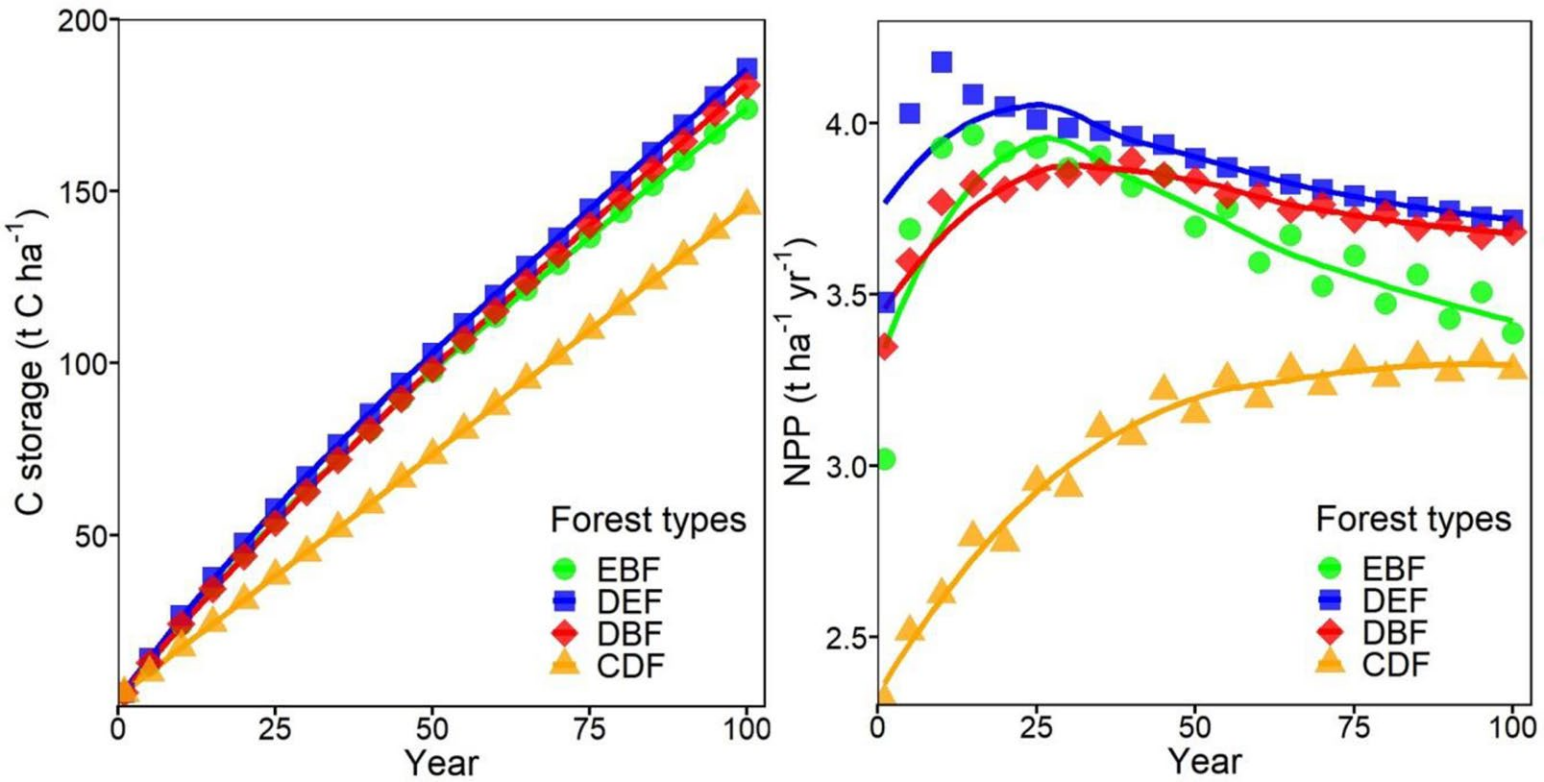
Changes in the predicted stand C storage (t C ha^−1^) and NPP (t ha^−1^ yr^−1^) by the TRIPLEX1.6 model among subtropical secondary forests over time (within 100 years). The markers (circle, square, diamond, and triangle) are the average values of simulated stand C storage and NPP at 5 year interval for each forest type of all plots under current climate conditions. The lines are the change patterns fitted by the predicted values for each forest type over 100 years. *EBF* evergreen broad-leaved forest, *DEF* deciduous and evergreen broad-leaved mixed forest, *DBF* deciduous and evergreen broad-leaved forest, *CDF* coniferous and broad-leaved mixed forest

Simulations and predictions of TRIPLEX1.6 revealed the dynamics of stand C storage and NPP among secondary forest types over 100 years (Fig. 2). Overall, the results showed that there were significant differences in stand C storage and NPP between CDF and the three other subtropical secondary forests. Stand C storage gradually increased in all forests in 100 years, whereas the stand C storage of CDF was significantly lower than that of the other three forests. The dynamics of stand NPP revealed a difference in the growth rate of C storage among secondary forests. The NPP of three broad-leaved forests peaked in approximately 25 years and then decreased gradually over time, whereas the NPP of CDF increased for 50 years and then stabilized. The NPP was significantly lower in CDF than in the other three forests.

**Fig. 3:**
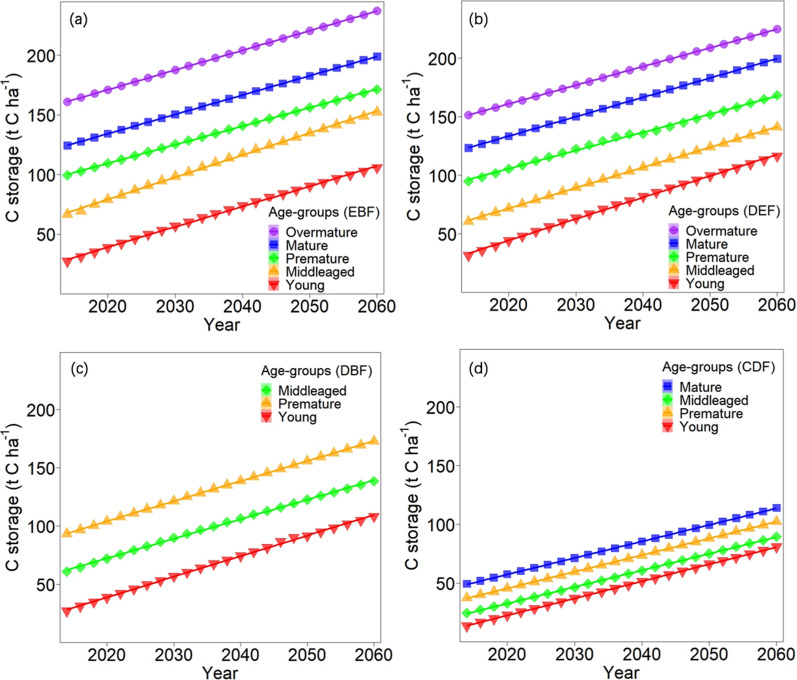
Changes in predicted stand C storage (t C ha^−1^) for different age-groups of subtropical secondary forests from 2014 to 2060: **a** evergreen broad-leaved forest; **b** deciduous and evergreen broad-leaved mixed forests; **c** deciduous broad-leaved forest; and **d** coniferous and broad-leaved mixed forest. The markers are the average values of predicted stand C storage at one year interval for each age-group of four forest types of all plots from 2014 to 2060. The lines are the change patterns fitted by the predicted values for each age-group of four forest types

**Fig. 4:**
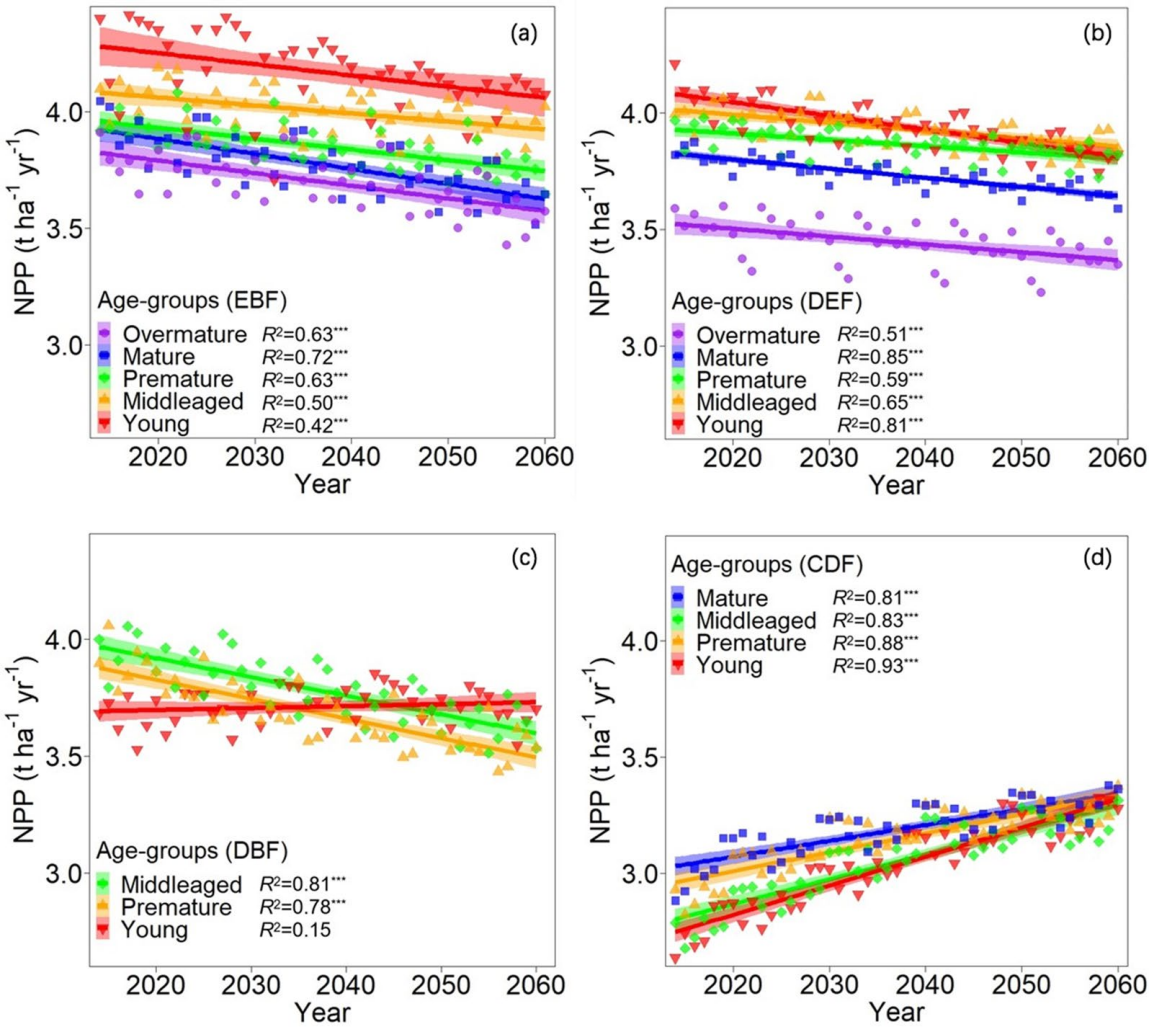
Change in predicted NPP (t ha^−1^ yr^−1^) for different age-groups of subtropical secondary forests from 2014 to 2060: **a** evergreen broad-leaved forest; **b** deciduous and evergreen broad-leaved mixed forests; **c** deciduous broad-leaved forest; and **d** coniferous and broad-leaved mixed forest. The markers are the average values of predicted NPP for each age-group of four forest types of all plots from 2014 to 2060. Solid regression lines are the significant relationship fitted by the predicted values for each age-group of four forest types, with 95% confidence intervals indicated by shading

As the forest develops, the age composition will change over time. Most forests gradually change from young to mature and over-mature. We then predicted the C storage and NPP among secondary forests across the age groups from 2014 to 2060 (Figs. 3 and 4). C storage of over-mature forest was consistently the highest in all age groups among the different forest types, and the C storage across all age groups gradually increased. The NPP of young forest in the three broad-leaved forests was the highest in all age groups and then decreased over time (the young forest in DBF could not be well fitted); the NPP of mature forest in CDF was the highest and then increased over time.

### Effects of climatic change on stand C storage and NPP in secondary forests

We predicted stand C storage and the difference in NPP between current and future climate scenarios (RCP4.5 and RCP8.5) among secondary forests (Table 1). We found that future climate changes were predicted to result in increased stand C storage and NPP for all subtropical forests. Overall, the response under RCP8.5 and RCP4.5 was the same. Under the same climate scenario, the response of NPP in forests in 2030 was stronger than that in 2060. The response of stand C storage and NPP of the four forest types under the climate scenarios differed. The difference in stand C storage for CDF was significantly lower compared with that for the other three forests (*p* < 0.001) (Table 1). The difference in NPP for EBF and CDF was significantly lower than that for DBF and DEF (*p* < 0.001) (Table 1).

**Table 1:** The differences in C storage (t C ha^−1^) and NPP (t ha^−1^ yr^−1^) (mean ± standard deviation) of four subtropical secondary forests calculated in 2030 and 2060 between current and the future climate scenarios (RCP4.5 and RCP8.5, *p* < 0.001)

Forest type	C storage in 2030		C storage in 2060		NPP in 2030		NPP in 2060	
	RCP4.5	RCP8.5	RCP4.5	RCP8.5	RCP4.5	RCP8.5	RCP4.5	RCP8.5
EBF	4.33 ± 0.45^a^	4.33 ± 0.45^a^	5.87 ± 0.57^a^	6.41 ± 0.63^a^	0.13 ± 0.02^b^	0.14 ± 0.02^b^	0.09 ± 0.01^b^	0.10 ± 0.01^b^
DEF	4.47 ± 0.78^a^	4.47 ± 0.78^a^	6.81 ± 0.97^a^	7.54 ± 0.99^a^	0.20 ± 0.01^a^	0.22 ± 0.01^a^	0.15 ± 0.01^a^	0.17 ± 0.01^a^
DBF	4.62 ± 0.32^a^	4.62 ± 0.32^a^	7.31 ± 0.54^a^	8.13 ± 0.92^a^	0.20 ± 0.01^a^	0.22 ± 0.01^a^	0.17 ± 0.01^a^	0.18 ± 0.05^a^
CDF	2.11 ± 0.11^b^	2.11 ± 0.11^b^	3.71 ± 0.15^b^	4.15 ± 0.16^b^	0.10 ± 0.01^b^	0.12 ± 0.01^b^	0.10 ± 0.00^b^	0.11 ± 0.00^b^

Different letters in the same column indicate significant differences (*p* < 0.001), and the same letters indicate no significant differences

*EBF* evergreen broad-leaved forest, *DEF* deciduous and evergreen broad-leaved mixed forest, *DBF* deciduous broad-leaved forest, *CDF* coniferous and broad-leaved mixed forest

### Prediction of the stand C storage and NPP of secondary forests in 2030 and 2060

Overall, the average stand C storage of subtropical secondary forests is expected to reach 65.74 t C ha^−1^ in 2030 and 113.71 t C ha^−1^ in 2060. To understand the role of the four subtropical secondary forests in reaching targets of having CO_2_ emissions peak in 2030 and achieving C neutrality in 2060, stand C storage and NPP of the four forest types under the three climate scenarios (current, RCP4.5, and RCP8.5) were predicted (Table 2). Under all climate scenarios, stand C storage was the highest in EBF and smallest in CDF. The growth rate of C storage was the fastest in DEF from 2014 to 2030 and in CDF from 2030 to 2060. NPP was highest in DEF and lowest in CDF.

**Table 2:** C storage (t C ha^−1^), C storage growth rate (%), and NPP (t ha^−1^ yr^−1^) in the four subtropical secondary forests predicted under current and future climate scenarios (RCP4.5 and RCP8.5) in 2014, 2030, and 2060

Forest type	Climate scenario	2014		2030			2060		
		C storage	NPP	C storage	C storage growth rate (%)	NPP	C storage	C storage growth rate (%)	NPP
EBF	Current	55.50 ± 41.57	3.86 ± 1.36	85.22 ± 44.47	53.55	3.83 ± 1.23	135.76 ± 51.01	59.31	3.59 ± 0.80
DEF		41.09 ± 32.69	4.09 ± 1.87	77.34 ± 25.16	88.22	3.95 ± 0.25	129.57 ± 24.95	67.53	3.81 ± 0.24
DBF		40.37 ± 27.41	3.87 ± 1.47	69.21 ± 34.58	71.44	3.78 ± 1.06	119.05 ± 46.53	72.01	3.75 ± 0.91
CDF		28.14 ± 15.60	2.36 ± 0.97	50.57 ± 21.24	79.71	3.04 ± 0.91	93.54 ± 29.35	84.97	3.25 ± 0.66
Average		37.12 ± 28.56	3.29 ± 1.29	65.74 ± 31.21	77.10	3.54 ± 0.97	113.71 ± 38.70	72.97	3.54 ± 0.69
EBF	RCP4.5	–	–	88.56 ± 47.29	59.57	3.91 ± 1.13	138.63 ± 54.15	62.67	3.68 ± 0.81
DEF		–	–	81.81 ± 25.50	99.10	4.15 ± 0.24	136.38 ± 25.32	76.34	3.96 ± 0.24
DBF		–	–	74.88 ± 38.21	85.48	4.03 ± 1.41	129.36 ± 51.54	86.91	3.92 ± 1.00
CDF		–	–	52.69 ± 22.96	87.24	3.15 ± 0.97	97.40 ± 31.71	92.60	3.35 ± 0.69
Average		–	–	69.26 ± 33.40	86.58	3.69 ± 1.05	119.27 ± 41.62	81.43	3.67 ± 0.73
EBF	RCP8.5	–	–	88.57 ± 47.57	59.59	3.92 ± 1.14	139.17 ± 54.46	63.31	3.69 ± 0.81
DEF		–	–	82.31 ± 25.76	100.32	4.16 ± 0.24	137.11 ± 25.62	77.28	3.98 ± 0.24
DBF		–	–	75.41 ± 38.63	86.80	4.05 ± 1.42	130.19 ± 52.11	88.11	3.90 ± 1.01
CDF		–	–	52.94 ± 23.11	88.13	3.16 ± 0.97	97.84 ± 31.90	93.47	3.36 ± 0.69
Average		–	–	69.65 ± 33.67	87.63	3.70 ± 1.05	119.87 ± 41.97	82.34	3.68 ± 0.74

*EBF* evergreen broad-leaved forest, *DEF* deciduous and evergreen broad-leaved mixed forest, *DBF* deciduous broad-leaved forest, *CDF* coniferous and broad-leaved mixed forest

## Discussion

### C sequestration capacity of subtropical secondary forests

The C sequestration capacity of subtropical secondary forests varies at different restoration stages. Predictions of stand C storage in 2060 by the TRIPLEX1.6 model indicated that stand C storage of EBF was greater than that of other forest types. Similar results were obtained in Jiangxi and Hunan [15, 32]. Previous studies that measured the biomass of permanent forest plots in subtropical forest [42, 43] have suggested that the above-ground biomass of subtropical old-growth EBF in China is 210–230 t ha^−1^ [44]. This value is consistent with our simulated values of C storage using a conversion coefficient of 0.5. These findings indicate that EBF plays an important role in forest C storage and productivity capacity in subtropical areas. The key role of these late restoration stage forests in maintaining C stocks by preventing emissions derived from deforestation, forest degradation, and future climate warming emphasizes the need for habitat protection and sustainable management of complex and heterogeneous subtropical secondary forests [3].

There were also differences in the rate of C production among forest types. The increasing trend of NPP in CDF with stand age indicated that there were interdependent or showed complementary environmental adaptations, leading to higher resource utilization efficiency; improved use of their individual niches might also explain why the NPP in the forest increased [45, 46]. These findings indicate that CDF have the potential to support high C productivity. However, in most secondary forests, C stocks typically increase rapidly during the initial phase of regeneration, and then decelerate over subsequent decades and even centuries as primary forest species gradually colonize the area and grow to maturity [47]. The maximum NPP of EBF occurred earlier compared with that of other forests because more productive forests show an earlier growth peak [48].

The study of C sequestration in secondary forests is particularly important given that the proportion of secondary subtropical forests is projected to continue to increase because of increases in anthropogenic and environmental disturbances [7, 47]. Previous research has shown that the greater amount of C in primary forest compared with secondary forest stems in large part to the difference in the number of large trees [47]. Hence, the natural regeneration of forests is the cheapest and technically easiest option for achieving targets of having CO_2_ emissions peak in 2030 and reaching C neutrality in 2060. Protecting woodland from fire and other human disturbances allows trees to return and forests to flourish; it also allows C stocks to build rapidly and reach the level of C storage of a mature forest in roughly 70 years [7]. The protection of subtropical natural secondary forest not only involves restoring it to the state of mature forest but also altering forest structure and composition so that it has a more stable C sequestration capacity similar to evergreen broad-leaved forest.

### The influence of stand age on stand C storage and NPP

Forest age was an important factor in modulating the vegetation C storage. Owing to nutrient limitation, stomatal constraint, declines in photosynthesis during the stand development, stand NPP decreased with stand age [14, 49]. Previous studies indicate that NPP generally decreases in old forests to about half or one-third of its maximum value; other researchers have found old forests to be as productive as young forest stands [15]. In our study, the predicted NPP of old forests (100 years) decreased by 7.77–16.50% from its peak for all forest types except for CDF. The continual recruitment of younger trees and rapid leaf area replacement, the compensatory growth of remaining vegetation, and canopy structural heterogeneity may offset this decrease in NPP in the moderately disturbed forests in this study [12]. These results indicate that subtropical secondary forests in China still have high potential for C sequestration compared with other forests, and make C storage will continue to increase with stand growth. Particularly, C storage is related to changes in the age composition [15]. These indicate that forest managers should pay more attention to the C sequestration contribution of old-growth forests.

### Responses of stand C storage and NPP in subtropical forests to climate change

We compared the stand C storage and NPP between future climate change (RCP4.5 and 8.5) and the current state in 2030 and 2060. Climate change had a positive effect on forest C storage, and this might be explained by the fact that higher water availability (precipitation) and heat (temperature) can promote vegetation productivity [20, 50]. Nevertheless, the C sequestration potential under future climate change was still limited in the short and medium-term. This response of C storage to climate change is consistent with previous studies [51–53]. Stand C storage and NPP significantly differed across forest types (*p* < 0.001). Climate change responses of forests were more sensitive in the middle of recovery, and this result is consistent with Wu et al. [53]. This might be driven by species-specific physiological characteristics and site conditions [41, 54]. Under disturbances or environmental changes, species asynchrony, facilitation, and species interactions might be the main drivers of ecosystem stability [11, 55]. Differences in the response of forest types and species to climate change contribute to forest community composition. Hence, understanding these differences would aid the development of measures for mitigating the negative effects of climate change, such as changes in stand structural attributes [53].

### Model evaluation, limitations, and uncertainty

We calibrated and validated the TRIPLEX1.6 model with 875 permanent forest plot records in 2014 to obtain the optimal parameters for EBF, DEF, DBF, and CDF, which are four types of subtropical natural secondary forests in southern China. Our focus in adjusting model parameters was on the initial value of soil parameters. Soil fertility is an important initial input variable [20]. The soil fertility should be set based on the growth of the forest, the thickness of the humus layer observed in the field, and the degree of stress that the soil has experienced in the past. The value of soil fertility in same area are provided in previous studies, however, subtropical ecosystems are vulnerable to extreme climate events caused by global warming [56]. For example, the heavy snow/ice event that occurred in 2008 in southern China severely damaged forest ecosystems and resulted in decreased soil fertility. Therefore, the effect of historical natural disasters should be considered when setting the initial value of soil fertility. High consistency between the observed and simulated data from the model was achieved via model parameterization and repetitious adjustments.

Our prediction of C storage indicates that it will accumulate gradually with forest growth in medium-term, and changes in NPP among subtropical secondary forests were consistent with those reported in previous studies [15, 57]. All stand variables had *R*^*2*^ values of 0.87–0.99, which indicated that they could be used to predict growth C dynamics across various stand ages. These findings of model performance are consistent with previous studies showing that the TRIPLEX1.6 model is robust and can be used to predict the growth of subtropical natural secondary forests and artificial forests, including *C. lanceolata* and *P. massoniana* in southern and southeast China with *R*^*2*^ values of 0.94 for total biomass C [38] and 0.91 for total biomass [39]. Combined with previous simulation results of coniferous forests, the TRIPLEX1.6 model could simulate forest growth with a high level of accuracy for all forest types in subtropical China.

The high biophysical heterogeneity and large amount of young age group subtropical secondary forests in subtropical China have increased the difficulty of evaluating the role of stand C storage and NPP in subtropical forests [57, 58]. Because of data limitations, the renewal of natural forest and under story regeneration in secondary forests was not considered in this study, which might reduce the robustness of our simulation results. Subtropical secondary forest has a high C sequestration capacity. In this study, the existing area of vegetation was not considered. Accurate prediction of spatial distributions is a major challenge and an important topic that should be examined in future studies. TRIPLEX1.6 simulations do not consider several variables related to contingency factors (i.e., anthropogenic disturbances, infestations, and extreme weather), which decreases their accuracy [37]. Despite these uncertainties, the TRIPLEX1.6 model suggests that the secondary forests in subtropical China have a high capacity for C sequestration and storage, but the effects of climate change (RCP4.5 and RCP8.5) on stand C storage and NPP differ among forest types.

## Conclusions

We used inventory plots data at different restoration stages and hybrid model of TRIPLEX 1.6 to predict changes in stand C storage and NPP under the two climate change scenarios (RCP4.5 and RCP8.5). Stand C storage of EBF was predicted to be the largest, and the NPP of DEF was predicted to be the highest. The NPP in CBF increased gradually over time and showed strong C storage potential capacity. Old-growth forests (mature and over-mature forests) have an important role in forest C sequestration. We not only considered the impact of stand age and forest types on C storage and NPP, but also made predictions about the impact of climate change under carbon neutrality in 2060. There was a significant difference in the effects of climate change (RCP4.5 and RCP8.5) on stand C storage between CDF and the other three forest types, but these changes were still limited in short and medium-term. These results indicate that the role of the floristic composition and tree growth of existing forests should be considered by forest managers for increasing C sequestration in secondary forests.

## Methods

### Study area

**Fig. 5:**
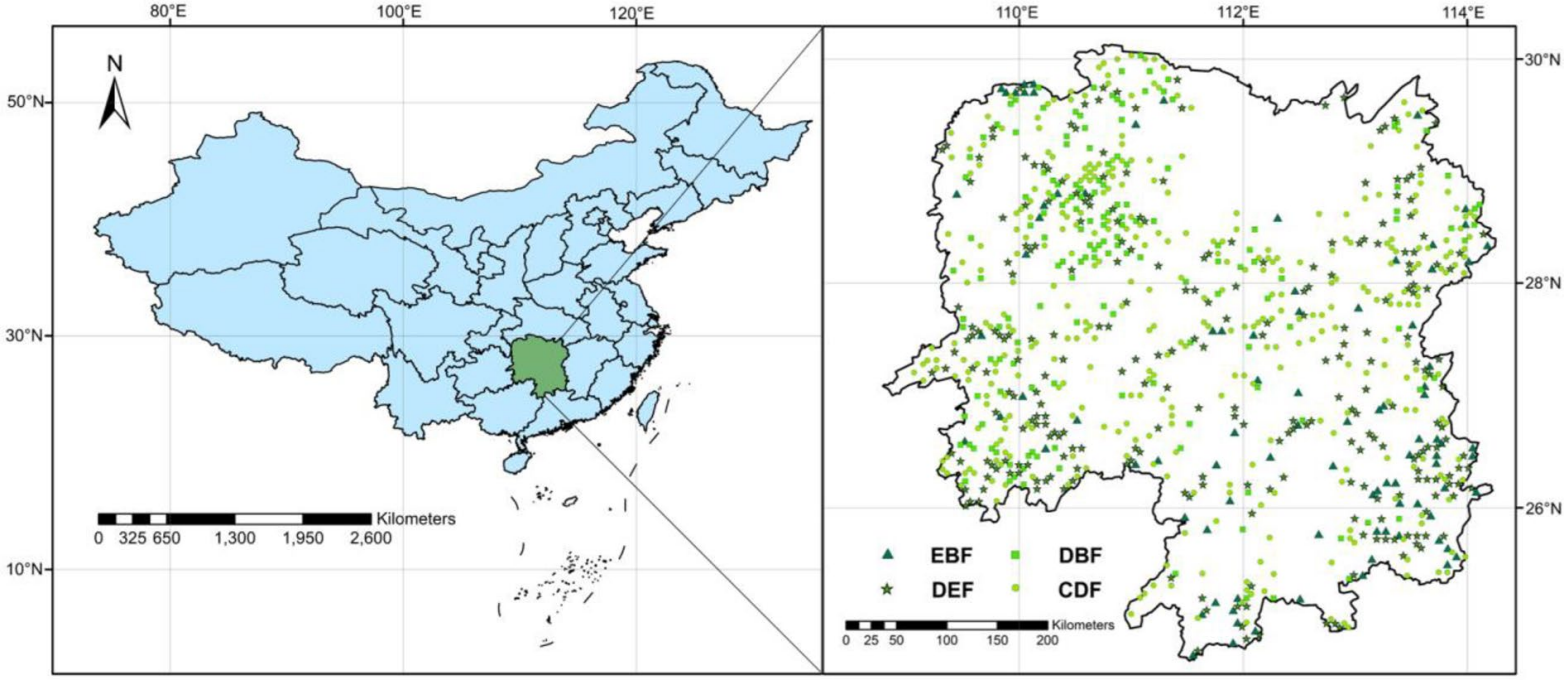
The spatial distribution of 875 permanent sample plots (PSP) with 93 PSP in evergreen broad-leaved forest (EBF), 267 PSP in deciduous and evergreen broad-leaved mixed forest (DEF), 155 PSP in deciduous broad-leaved forest (DBF), and 360 PSP in conifer and broad-leaved mixed forest (CDF) in Hunan Province, southern China, respectively (modified in 2014)

This study was carried out in Hunan Province (24°38′–30°08′ N and 108°47′–114°15′ E, Fig. 5) in central subtropical China. This province features mountains in the east, south, and west with elevations ranging from 21.60 to 2115.20 m (Fig. 5), hills in the east-central region, and plains in the northeast region surrounding Dongting Lake. The topography is heterogeneous, with slopes between 0° and 70°. The total land area is 21.18 million ha, including 12.53 million ha of forests, 3.92 million ha of arable land, 1.20 million ha of land covered by water bodies, and 1.00 million ha of developed land.

**Fig. 6:**
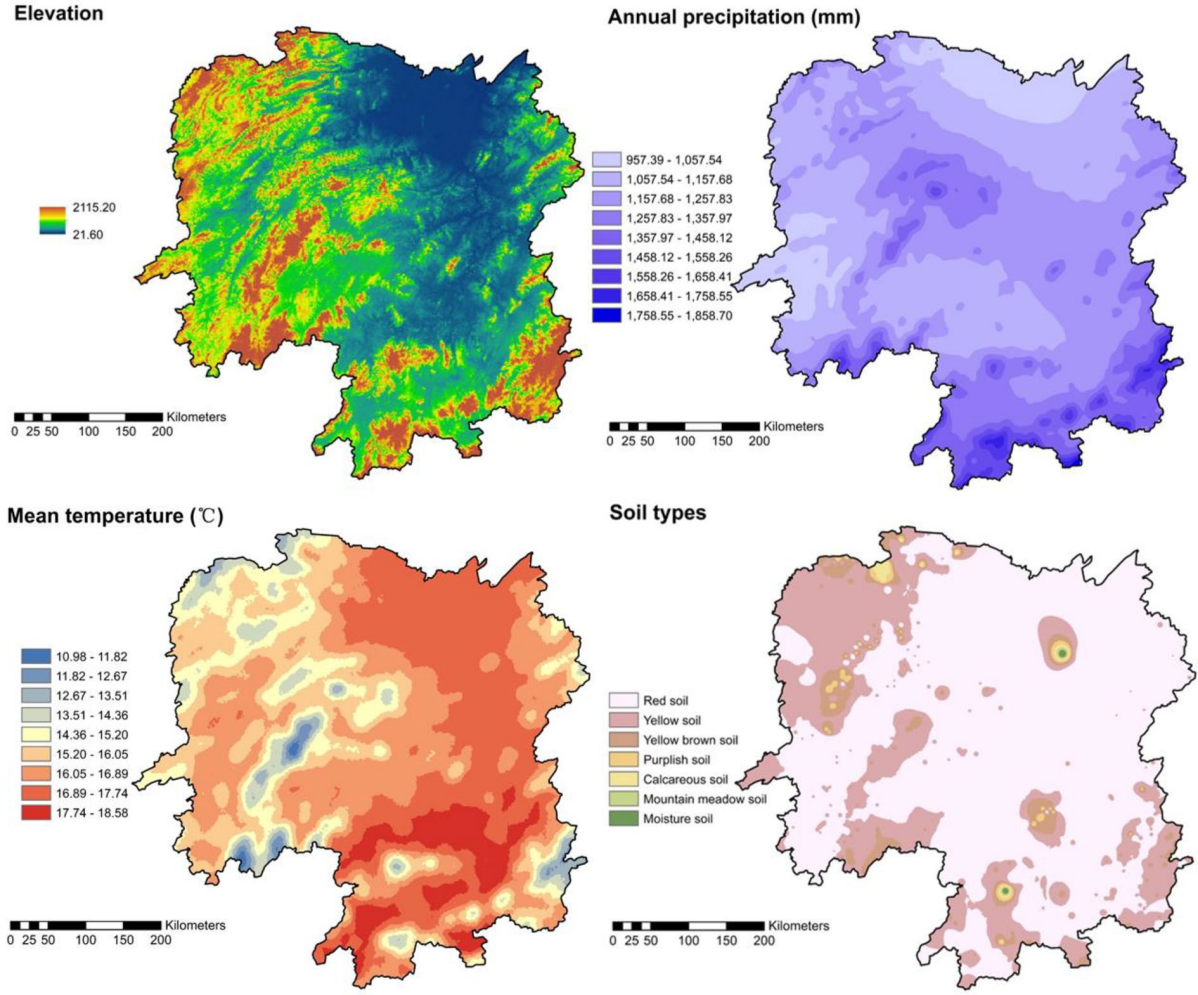
Maps showing variation in elevation, annual precipitation, annual average temperature, and soil types in Hunan Province

This region has a continental and subtropical humid monsoon climate that is rich in light, heat, and water resources (Fig. 6). The mean annual rainfall is 1500 mm, with 75% falling between March and August. The mean annual temperature is 14.10 °C. Annual climate changes are large: summers are hot, and winters are short and cold. The soil types include red soil, yellow soil, and red-yellow soil, with small areas of calcareous soil, purple soil, moisture soil, and mountain meadow soil (Fig. 6), which are mainly developed from granite, limestone, and shale.

### Forest types and stand age groups

The National Forest Inventory of China classified subtropical forests into four forest types according to the dominant tree species: pine forest, fir forest, deciduous forest, and evergreen broad-leaved forest [59]. These forest types encompass most but not all wide spread tree species in Hunan. DEF are an important forest type in the restoration process that have not been examined extensively in previous studies because of the difficulty of determining the dominant tree species and stand structure. Thus, we divided subtropical secondary forest into four forest types: CDF, DBF, DEF, and EBF. Forest type identification was based on the technical regulations for continuous forest inventory: coniferous or broad-leaved species accounted for less than 70% of the species in CDF; the proportion of broad-leaved trees accounted for more than 70% of the species in broad-leaved forest; and the proportion of all broad-leaved species accounted for less than 70% of the species in DEF [60]. Among the natural secondary forests in Hunan, CDF are mainly dominated by *Pinus massoniana* forests; the DBF are mainly dominated by *Alniphyllum fortunei*, *Choerospondias axillaris*, and *Liquidambar formosana.* Hance; and tree species in the EBF are mainly dominated by *Cyclobalanopsis glauca* Thunb, *Litsea coreana* Levl. var. *sinensis*, and *Schima superba* Gardn.

The study area is composed of subtropical natural uneven-aged forest. Therefore, five of the largest trees outside each plot were selected for stem core sampling, and the ring counts of these five tree samples were averaged to estimate stand age [13]. According to the forestry standards of China, the age composition of four forest types can be divided into young forest, middle-aged forest, premature forest, mature forest, and over-mature forest. The age group of the two mixed forests was determined by the age group of the dominant tree species and their growth condition [An additional table file shows this in more detail (see Additional file 1)].

### TRIPLEX1.6 model description

TRIPLEX is a hybrid model [An additional table file shows this in more detail (see Additional file 2)] that integrates three well-established process-based sub-models: the forest production sub-model 3-PG [61], the forest growth and yield sub-model TREEDYN3.0 [33], and the soil-C, soil-N (nitrogen) and soil–water-balance sub-model CENTURY4.0 [62]. The forest production sub-model estimates monthly GPP (including above- and below-ground biomass) from photosynthetic active radiation (PAR), mean air temperature, vapor pressure deficit (VPD), soil water, the percentage of frost days, and the leaf area index. The forest growth and yield sub-model calculates tree growth and yield variables (height, DBH) using a function of the stem wood biomass increment [33]. The soil C, soil N, and soil–water balance sub-model simulate soil C and N dynamics between litter and soil pools, simulate water balances and dynamics, and calculate monthly water loss through transpiration, evaporation, soil water content, and snow water content [62]. TRIPLEX combines empirical and mechanistic components that can simulate various growth volumes in forest ecosystems using input data derived from site, soil, climate, and stand growth [36]. The hybrid model compensates for the inability of simplified growth models to take into account soil factors, feedback between the forest ground and underground, and climate impacts. The simulation of the TRIPLEX model involves key processes and dynamics including PAR, GPP, forest growth, biomass, soil C, soil nitrogen, and soil water [40]. After the model was optimized to version 1.6, a reasonable and balanced parameter generalization procedure that did not lead to a significant reduction of model accuracy but increased model practicability was described [40].

The TRIPLEX1.6 simulation requires input data such as latitude, longitude, soil texture, monthly climate records, tree species physiological variables (such as maximum tree height and diameter), tree species process mediators, stand structure, and certain initial site conditions. Simulation outputs include tree diameter, height, basal area, total volume, leaf area index, GPP, net ecosystem production (NEP), biomass, soil C, and N and water dynamics. TRIPLEX1.6 has been tested and applied to forest growth and biomass production on a regional scale at Zhejiang Province, China [39, 63] and northeastern China [37] and has also been used to predict *Cunninghamia lanceolata* and *P. massoniana* stand production in Hunan Province, China [38]. Natural secondary forests in a subtropical region were examined in this paper, and the suitability of the model in the subtropical region has been demonstrated by previous studies [38].

### Model input data

There were five primary data sources: permanent forest-plot (667 m^2^) records taken from the forest inventory, climate datasets, field observations, the literature, and assumptions. In Hunan Province, there were 875 permanent sample plots of natural secondary forest in 2014. The permanent forest plot database is a well-designed forest inventory program built from data acquired at 5-year intervals beginning in 1973. Field investigations in the permanent forest plot database include records of location, site conditions, and DBH of each tree; average stand height [64]; and land use, site class, dominant tree species, stand density, age, average DBH, average tree height, and volume [65]. Plots included at least one of the four forest types and met the criteria for tree size (e.g., DBH ≥ 5.0 cm) and stage of stand development (with the exception of seedlings). Plots were required to meet various criteria regarding site, climate, and growth, and the initial data needed to be detailed and representative of the forest types, as well as of the study area as a whole [38]. According to the forest plot database, stand biomass can be estimated using allometric equations [46] [An additional table file shows this in more detail (see Additional file 3)] for the main subtropical tree species. The conversion parameter between biomass and C storage was 0.5. Based on data collected in 2009 and 2014, we calculated average annual NPP (t ha^−1^ year^−1^) using the following formula:

1 $$NPP = \frac{B_{t2} - B_{t1}}{{t_2 - t_1}}$$

where *B*_*t1*_ and *B*_*t2*_ are the plot biomass in year t_1_ (2009) and t_2_ (2014), respectively. Climate data for each permanent plot were interpolated from data collected from meteorological stations [38]. The data recorded between 2005 and 2014 were used as climate inputs for each plot. The climate inputs were monthly frost days, monthly average air temperature, monthly sum of precipitation, and the monthly average atmospheric VPD [36]. Atmospheric N deposition was set to 18 kg N ha^−1^ year^−1^ [59]. Field observations were made at independent sampling plots in Hunan Province. The choice of reference literature and assumptions were based on indicator functions for the four forest types selected for this study [66–68].

Predictors for the TRIPLEX1.6 model were climate normal from the 96 automatic meteorological monitoring stations in Hunan Province for current (2005–2014) and future (2015–2060) periods. The future climate change scenario data were derived from the WCRP’s CMIP5 multi-model dataset provided by the National Climate Center of China Meteorological Administration (http://www.climatechange-data.cn). According to the fifth report of the IPCC, we obtained data for current scenarios, RCP4.5 (medium emissions scenario, assumes the imposition of emissions mitigation policies), and RCP8.5 (high emissions scenario, does not include any specific climate mitigation target) to simulate the responses of different forest types and age composition to future climate change. Because the CMIP5 GCMs have different horizontal resolutions, future climate data were interpolated on a common 1° × 1° grid using the nearest neighbor interpolation method.

### Model initialization, parameterization, and simulations

From the initial modelling, we examined the most sensitive parameters through direct measurement, literature searches, default values, and statistical fitting. To simulate forest ecosystem processes and dynamics, TRIPLEX1.6 requires some initial values of stand variables describing conditions of forest stands and soils. There are three key variables (stand density, tree height, and DBH) related to initial conditions for forest growth and yield simulation [40]. To ensure the robustness of the TRIPLEX1.6 model, most of the general and nonspecific site parameters were derived from previous studies. These include PAR parameters; the minimum, maximum, and optimum temperature for tree growth; stomata and canopy conductance; initial N for tree growth; the lignin-nitrogen ratio and lignin fraction of leaf and fine and coarse roots; and the fraction of soil water flow [39]. Several new parameters such as wood C density, specific leaf area (SLA), mortality, the fraction of leaf, branch, wood, and coarse and fine roots were used and adjusted from default model values to better represent the forest ecosystems of subtropical China for this study [An additional table file shows this in more detail (see Additional file 4 and 5)].

TRIPLEX1.6 was calibrated by randomly selecting one-third of each forest type and validated by remainder data before simulation runs. The simulation was executed for DBH, stem density, C storage, and NPP. We simulated each stand from its respective year of regeneration to 2014, at which point all simulations across all stands within the natural subtropical forest region were summed. The same procedure was followed for all model runs by Zhou et al. [40].

## Supplementary Information

**Additional file 1.** Age group classification standard of subtropical natural secondary forests in Hunan Province.

**Additional file 2.** The structural model of forest growth and carbon simulation from TRIPLEX1.0 (modified from Peng et al., 2002). Rectangles represent key pools or state variables, ovals represent core simulation processes, dotted lines represent controls, and solid lines represent the flow of carbon (C), nitrogen (N), water, and the fluxes between the forest ecosystem and external environment. Two arrow cycles refer to two feed-backs.

**Additional file 3.** Compatibility superposition relative growth equation (ln*W*_i_ = *ß*_i0_ + *ß*_i1_ × ln*D* ) regression coefficients of biomass ( *W*_i_, kg) and DBH (*D*, cm) for each part of the tree species (values in parentheses is standard error) and fitting parameters.

**Additional file 4.** Site-specific parameters used in TRIPLEX1.6 for simulating subtropical forest ecosystems in Hunan Province, subtropical China (Zhao et al., 2013).

**Additional file 5.** Stand growth and tree species-specific parameters TRIPLEX1.6 applied to simulate growth of evergreen broad-leaved forest, deciduous and evergreen broad-leaved mixed forest, deciduous broad-leaved forest, and coniferous and broad-leaved mixed forest.

**Additional file 6.** Descriptive statistics for the spatial heterogeneity of stand growth by forests in all selected permanent forest plots in Hunan Province in 2014. Including stand-structure variables: stem density (stems ha^-1^), stand age (year), diameter at breast height (DBH, cm), height (m), NPP (t ha^-1^ yr^-1^) and stand biomass (t ha^-1^).

**Additional file 7.** Descriptive statistics for spatial heterogeneity of climate and forest stand structure in all selected permanent forest sample plots in Hunan Province. Site variable: elevation (above mean sea level, m); climatic variables: annual average air temperature (°C), annual rainfall (mm yr^-1^) and the annual average vapor pressure deficit (mbar) between 2000 and 2014.

**Additional file 8.** Simulation errors of TRIPLEX1.6 applied to subtropical forest ecosystems in southeastern China, comparing density (stems ha−1), DBH (cm), NPP (t ha-1 yr-1) and C storage (t C ha−1) between modeled values and forest inventory data collected from 875 forest stands.

**Additional file 9.** Variation of monthly average temperature and precipitation in Hunan Province from 2000 to 2014.

**Additional file 10.** Variation of monthly average temperature and precipitation in Hunan Province from 2000 to 2014.

## Data Availability

All data parameters about the TRIPLEX1.6 model on all of the conclusions of the manuscript rely to be presented in the additional supporting files (see Additional files 6 and 7). And the statistical parameters of sample plot data and climatic conditions and calculation method of forest vegetation carbon are also mentioned in the additional supporting files (see Additional files 8, 9, and 10).

## Abbreviations

C: Carbon
N: Nitrogen
NPP: Net primary production
NEP: Net ecosystem production
GPP: Gross primary productivity
PAR: Photosynthetic active radiation
VPD: Vapor pressure deficit
DBH: Diameter at breast height (1.3 cm)
PSP: Permanent sample plots
EBF: Evergreen broad-leaved forest
DEF: Deciduous and evergreen broad-leaved mixed forest
DBF: Deciduous broad-leaved forest
CDF: Conifer and broad-leaved mixed forest
UNFCCC: UN framework convention on climate change
