# Supplementary material for: Stand carbon storage and net primary production in China’s subtropical secondary forests are predicted to increase by 2060
Source: Carbon Balance Manag. 2022 May 26;17:6. doi: 10.1186/s13021-022-00204-y (PMC9134694; doi:10.1186/s13021-022-00204-y)
Supplement: Supplementary file 1 — Additional file 1. Age group classification standard of subtropical natural secondary forests in Hunan Province. [file 13021_2022_204_MOESM1_ESM.doc]

**Additional file A.** Age group classification standard of subtropical natural secondary forests in Hunan Province.

| Forest types | Age group | | | | |
| --- | --- | --- | --- | --- | --- |
|  | Young forest | Middle aged forest | Premature forest | Mature forest | Over-mature forest |
| Evergreen broad-leaved forest | ≤20 | 21~40 | 41~50 | 51~70 | ≥71 |
| Deciduous and evergreen broad-leaved mixed forest | ≤20 | 21~40 | 41~50 | 51~70 | ≥71 |
| Deciduous broad-leaved forest | ≤20 | 21~40 | 41~50 | 51~70 | ≥71 |
| Conifer and broad-leaved mixed forest | ≤10 | 11~20 | 21~30 | 31~50 | ≥51 |
