# Supplementary material for: Stand carbon storage and net primary production in China’s subtropical secondary forests are predicted to increase by 2060
Source: Carbon Balance Manag. 2022 May 26;17:6. doi: 10.1186/s13021-022-00204-y (PMC9134694; doi:10.1186/s13021-022-00204-y)

**Additional file B.** The structural model of forest growth and carbon simulation from TRIPLEX1.0 (modified from Peng et al., 2002). Rectangles represent key pools or state variables, ovals represent core simulation processes, dotted lines represent controls, and solid lines represent the flow of carbon (C), nitrogen (N), water, and the fluxes between the forest ecosystem and external environment. Two arrow cycles refer to two feed-backs.


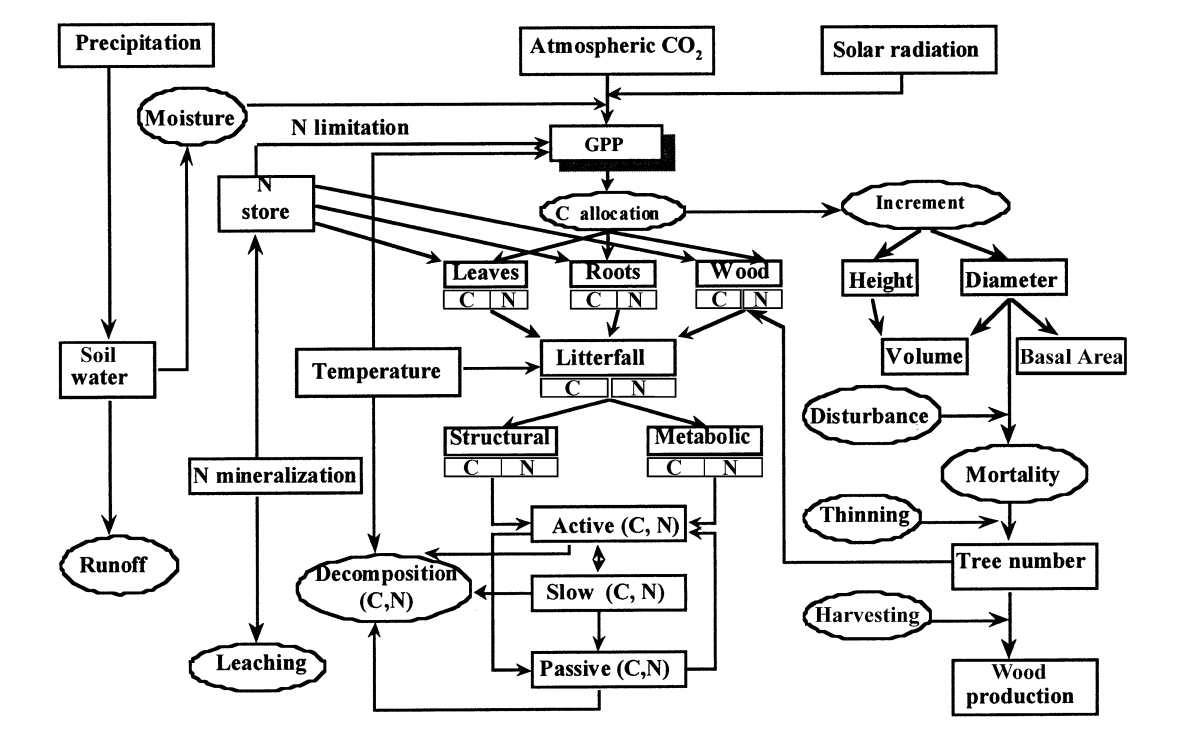

Supplement: Supplementary file 2 — Additional file 2. The structural model of forest growth and carbon simulation from TRIPLEX1.0 (modified from Peng et al., 2002). Rectangles represent key pools or state variables, ovals represent core simulation processes, dotted lines represent controls, and solid lines represent the flow of carbon (C), nitrogen (N), water, and the fluxes between the forest ecosystem and external environment. Two arrow cycles refer to two feed-backs. [file 13021_2022_204_MOESM2_ESM.doc]
