# Supplementary material for: Stand carbon storage and net primary production in China’s subtropical secondary forests are predicted to increase by 2060
Source: Carbon Balance Manag. 2022 May 26;17:6. doi: 10.1186/s13021-022-00204-y (PMC9134694; doi:10.1186/s13021-022-00204-y)
Supplement: Supplementary file 3 — Additional file 3. Compatibility superposition relative growth equation (lnWi = ßi0 + ßi1 × lnD ) regression coefficients of biomass ( Wi, kg) and DBH (D, cm) for each part of the tree species (values in parentheses is standard error) and fitting parameters. [file 13021_2022_204_MOESM3_ESM.doc]

**Additional file C.** Compatibility superposition relative growth equation () regression coefficients of biomass (, kg) and DBH ($D$, cm) for each part of the tree species (values in parentheses is standard error) and fitting parameters.

| Forest types | Organ | *β_i0_* | *P* | *β_i1_* | *P* | *R*^2^ | n | RMSE | CF | AIC |
| --- | --- | --- | --- | --- | --- | --- | --- | --- | --- | --- |
| Evergreen broad-leaved forest | Trunk | -2.331(0.197) | <0.0001 | 2.473(0.066) | <0.0001 | 0.980 | 30 | 0.277 | 1.039 | 12.263 |
|  | Branch | -3.851(0.334) | <0.0001 | 2.589(0.116) | <0.0001 | 0.838 | 30 | 0.780 | 1.356 | 74.347 |
|  | Leaf | -3.212(0.524) | <0.0001 | 1.825(0.177) | <0.0001 | 0.788 | 30 | 0.832 | 1.414 | 78.182 |
|  | Root | -3.119(0.328) | <0.0001 | 2.280(0.106) | <0.0001 | 0.934 | 30 | 0.497 | 1.131 | 47.282 |
|  | Above-ground |  |  |  |  | 0.973 | 30 | 0.327 | 1.055 | 18.04 |
|  | Total |  |  |  |  | 0.977 | 30 | 0.314 | 1.050 | 14.693 |
| Deciduous broad-leaved  forest | Trunk | -2.739(0.268) | <0.0001 | 2.496(0.093) | <0.0001 | 0.962 | 30 | 0.370 | 1.071 | 29.547 |
|  | Branch | -4.859(0.331) | <0.0001 | 2.688(0.118) | <0.0001 | 0.913 | 30 | 0.622 | 1.213 | 60.609 |
|  | Leaf | -6.581(0.760) | <0.0001 | 2.653(0.264) | <0.0001 | 0.734 | 30 | 1.165 | 1.971 | 98.374 |
|  | Root | -3.860(0.286) | <0.0001 | 2.471(0.098) | <0.0001 | 0.870 | 30 | 0.674 | 1.255 | 65.518 |
|  | Above-ground |  |  |  |  | 0.968 | 30 | 0.365 | 1.069 | 25.709 |
|  | Total |  |  |  |  | 0.951 | 30 | 0.450 | 1.107 | 37.366 |
| All tree species | Trunk | -2.374(0.170) | <0.0001 | 2.417(0.057) | <0.0001 | 0.964 | 70 | 0.354 | 1.065 | 57.601 |
|  | Branch | -4.477(0.268) | <0.0001 | 2.607(0.092) | <0.0001 | 0.817 | 70 | 0.855 | 1.441 | 180.912 |
|  | Leaf | -5.499(0.475) | <0.0001 | 2.404(0.159) | <0.0001 | 0.728 | 70 | 1.050 | 1.735 | 209.686 |
|  | Root | -3.799(0.231) | <0.0001 | 2.452(0.078) | <0.0001 | 0.897 | 70 | 0.600 | 1.197 | 131.226 |
|  | Above-ground |  |  |  |  | 0.950 | 70 | 0.418 | 1.091 | 77.734 |
|  | Total |  |  |  |  | 0.948 | 70 | 0.424 | 1.094 | 79.642 |

Note:*β_ij_*, regression coefficients; *R*^2^, coefficient of determination; n, number of stands; RMSE, root-mean-square error; CF, correction factor (
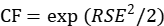
); AIC, Akaike’s information criterion.
