# Supplementary material for: Stand carbon storage and net primary production in China’s subtropical secondary forests are predicted to increase by 2060
Source: Carbon Balance Manag. 2022 May 26;17:6. doi: 10.1186/s13021-022-00204-y (PMC9134694; doi:10.1186/s13021-022-00204-y)
Supplement: Supplementary file 4 — Additional file 4. Site-specific parameters used in TRIPLEX1.6 for simulating subtropical forest ecosystems in Hunan Province, subtropical China (Zhao et al., 2013). [file 13021_2022_204_MOESM4_ESM.doc]

**Additional file D. Site-specific parameters used in TRIPLEX1.6 for simulating subtropical forest ecosystems in Hunan Province, subtropical China (Zhao *et al.*, 2013).**

| Meaning/comments | Name | Units | Value | Sources* |
| --- | --- | --- | --- | --- |
| *Canopy processes* |  |  |  |  |
| Extinction coefficient for absorption of PAR by canopy | ExtCoef | - | 0.5 | D |
| Canopy quantum efficiency | AlphaC | molC/molPAR | 0.033 | [1] |
| Damping factor, soil temperature | TemSF | - | 0.367 | [2] |
| Cloud ratio for a month | Cloud | - | 0.4 | O |
| Atmospheric absorption factor | Absorp | - | 0.089 | [3] |
| Solar radiation fraction | PARfactor | - | 0.829 | [3] |
| Fraction of H_2_O flow to stream | KF |  | 0.5 | D |
| Coefficient for effect of canopy conductance to VPD | Kg | - | 0.05 | D |
| *Conversion factors* |  |  |  |  |
| Power for deriving GPP | Na | - | 4 | D |
| *Temperature modifier* |  |  |  |  |
| Min temperature for producing GPP | TaMin | °C | 1 | O |
| Max temperature for producing GPP | TaMax | °C | 40 | O |
| Temperature of vegetation beginning and ending | Tveg | °C | 1 | O |
| Optimum temperature for producing GPP | Topt | °C | 17.5 | O |
| *Soil modifier* |  |  |  |  |
| Sand content rate (2-0.05mm %) | Ts | - | 0.16~0.18 | [4] |
| Clay content rate (<0.002mm %) | Tc | - | 0.4~0.42 | [4] |
| Silt plus clay content (0.05-0.002mm %) | T | - | 0.42~0.45 | [4] |
| Relative root density (layer 1) | AwL1 |  | 0.5 | [5] |
| Relative root density (layer 2) | AWL2 |  | 0.3 | [5] |
| Relative root density (layer 3) | AWL3 |  | 0.2 | [5] |
| Relative soil water content. 1.0 means flood | MoisRatio | - | 0.21~0.255 | [4] |
| SW constants | SWConst | - | 0.7 | [6] |
| Powers in the eqn. for SW modifiers | SWpwr | - | 9 | [6] |
| Fraction of H_2_O flow to deep storage | KD |  | 0.5 | D |
| Fraction of deep storage water to stream | KX |  | 0.3 | D |
| Maximum soil water | L_SoilWater | mm | 550.9 | [7] |
| *Fertility effects* |  |  |  |  |
| Nitrogen Factor | NitrogenFactor | - | 0.2 | D |
| Lignin-nitrogen ratio from N Module | Lnr |  | 0.26 | D |
| *Root turnover* |  |  |  |  |
| Leaf decomposition rate | LsLeaf | - | 0.215 | D |
| Root decomposition rate | LsRoot | - | 0.215 | D |
| Fine branch decomposition rate | LsFBranc | - | 0.235 | D |
| Wood decomposition rate | LsWood | - | 0.255 | D |
| Coarse root decomposition rate | LsCRoot | - | 0.255 | D |

* Sources: D = Default; O = Observed.

[1] Zhao, M.F., Xiang, W.H., Peng, C.H., Tian, D.L., 2009. Simulating age-related changes in carbon storage and allocation in a Chinese fir plantation growing in southern China using the 3-PG model. *Forest Ecology and Management*, 257, 1520-1531.

[2] Kang, W.X., Zhao, Z.H., Deng, X.W., 2007. Study of the dynamic effects and the law of kinetic energy transmission in the canopy of Chinese fir plantation ecosystems. *Journal of Central South University of Forestry & Technology*, 27, 1-6.

[3] Liu, X.Z., Kang, W.X., Wen, S.Z., 1993. Studies on energy balance on canopy in a Chinese fir plantation. *Long-term Located Research on Forest Ecosystem. Beijing: China Forestry Publish House*, 221-227.

[4] Chen, R., Xiang, W.H., Xu, X., Tian, D.L., Liu, J., 2010. Speed of soil nitrogen mineralization and effect of fertilizer combined with warming on it in different ages Chinese fir plantations. *Hunan Agricultural Sciences*, 5, 125-129.

[5] Zhao, K., Tian, D.L., 1999. Study of the biomass and productivity of mature Chinese fir stand in Huitong County. *Journal of Central-South Forestry College*, 20, 7-13.

[6] Zhao, M.F., Xiang, W.H., Deng, X.W., Tian, D.L., Huang, Z.H., Zhou, X.L., Yu, G.R., He, H.L., Peng, C.H., 2013. Application of TRIPLEX model for predicting *Cunninghamia lanceolata* and *Pinus massoniana* forest stand production in Hunan Province, southern China. *Ecological Modelling*, 250, 58-71.

[7] Tian, D.L., Kang, W.X., 1993. Study on the Chinese fir plantation adjustment function to water cycle. *Long-term Located Research on Forest Ecosystem. Beijing: China Forestry Publish House*, 203-208.
