# Supplementary material for: Stand carbon storage and net primary production in China’s subtropical secondary forests are predicted to increase by 2060
Source: Carbon Balance Manag. 2022 May 26;17:6. doi: 10.1186/s13021-022-00204-y (PMC9134694; doi:10.1186/s13021-022-00204-y)
Supplement: Supplementary file 5 — Additional file 5. Stand growth and tree species-specific parameters TRIPLEX1.6 applied to simulate growth of evergreen broad-leaved forest, deciduous and evergreen broad-leaved mixed forest, deciduous broad-leaved forest, and coniferous and broad-leaved mixed forest. [file 13021_2022_204_MOESM5_ESM.doc]

**Additional file E.** Stand growth and tree species-specific parameters TRIPLEX1.6 applied to simulate growth of evergreen broad-leaved forest, deciduous and evergreen broad-leaved mixed forest, deciduous broad-leaved forest, and coniferous and broad-leaved mixed forest.

| Meaning/comments | Name | Units | Value | Source* |
| --- | --- | --- | --- | --- |
| Evergreen broad-leaved forest | | | | |
| Specific leaf area | Sla | m^2^ kg^-1^ | 9.2±0.9 | [1] |
| Convert GPP->Npp | Ccpp |  | 0.495 | [1] |
| Wood C density | CSP | tCm^-3^ | 0.3648 | [1] |
| Crown to stem diameter ratio | CD | - | 20 | [2] |
| coef. For NPP allocation | Stemprn | - | 3 | E |
| coef. For NPP allocation | Stempra | - | 0.000006 | E |
| coef. For NPP allocation | Folprn | - | 2 | E |
| coef. For NPP allocation | Folpra | - | 0.0006 | E |
| Below ground wood fraction | RamdaU | - | 0.38 | [1] |
| Fine branch fraction | RamdaB | - | 0.22 | [1] |
| Min height-diameter | HdMin | - | 80 | [2] |
| Max height-diameter | HdMax | - | 120 | [2] |
| Max foliage | MaxGam |  | 0.03 | E |
| AgeMax | AgeMax | years | 120 | E |
| Root loss ratio | GamaR | - | 0.0054 | D |
| Normal mortality ratio | MiuNorm | - | 0.005 | D |
| Crowding mortality ratio | MiuCrowd | - | 0.01 | D |
| Stem loss ratio | GamaS | - | 0 | D |
| Deciduous broad-leaved forest | | | | |
| Specific leaf area | Sla | m^2^ kg^-1^ | 12.4±5.4 | E |
| Convert GPP->Npp | Ccpp |  | 0.495 | [1] |
| Wood C density | CSP | tCm^-3^ | 0.348 | [3] |
| Crown to stem diameter ratio | CD | - | 20 | [2] |
| coef. For NPP allocation | Stemprn | - | 3 | E |
| coef. For NPP allocation | Stempra | - | 0.000006 | E |
| coef. For NPP allocation | Folprn | - | 2 | E |
| coef. For NPP allocation | Folpra | - | 0.0006 | E |
| Below ground wood fraction | RamdaU | - | 0.36 | [1] |
| Fine branch fraction | RamdaB | - | 0.23 | [1] |
| Min height-diameter | HdMin | - | 80 | [2] |
| Max height-diameter | HdMax | - | 120 | [2] |
| Max foliage | MaxGam |  | 0.03 | E |
| AgeMax | AgeMax | years | 120 | E |
| Root loss ratio | GamaR | - | 0.0054 | D |
| Normal mortality ratio | MiuNorm | - | 0.005 | D |
| Crowding mortality ratio | MiuCrowd | - | 0.01 | D |
| Stem loss ratio | GamaS | - | 0 | D |
| Deciduous and evergreen broad-leaved mixed forest | | | | |
| Specific leaf area | Sla | m^2^ kg^-1^ | 15 | E |
| Convert GPP->Npp | Ccpp |  | 0.495 | [1] |
| Wood C density | CSP | tCm^-3^ | 0.3564 | E |
| Crown to stem diameter ratio | CD | - | 20 | [2] |
| coef. For NPP allocation | Stemprn | - | 3 | E |
| coef. For NPP allocation | Stempra | - | 0.000006 | E |
| coef. For NPP allocation | Folprn | - | 2 | E |
| coef. For NPP allocation | Folpra | - | 0.0006 | E |
| Below ground wood fraction | RamdaU | - | 0.37 | E |
| Fine branch fraction | RamdaB | - | 0.22 | E |
| Min height-diameter | HdMin | - | 80 | [2] |
| Max height-diameter | HdMax | - | 120 | [2] |
| Max foliage | MaxGam |  | 0.03 | E |
| AgeMax | AgeMax | years | 120 | E |
| Root loss ratio | GamaR | - | 0.0054 | D |
| Normal mortality ratio | MiuNorm | - | 0.005 | D |
| Crowding mortality ratio | MiuCrowd | - | 0.01 | D |
| Stem loss ratio | GamaS | - | 0 | D |
| Conifer and broad-leaved mixed forest | | | | |
| Specific leaf area | Sla | m^2^ kg^-1^ | 15 | [4] |
| Convert GPP->Npp | Ccpp |  | 0.490 | E |
| Wood C density | CSP | tCm^-3^ | 0.3082 | E |
| Crown to stem diameter ratio | CD | - | 20 | [2] |
| coef. For NPP allocation | Stemprn | - | 3 | E |
| coef. For NPP allocation | Stempra | - | 0.000006 | E |
| coef. For NPP allocation | Folprn | - | 2 | E |
| coef. For NPP allocation | Folpra | - | 0.0006 | E |
| Below ground wood fraction | RamdaU | - | 0.13 | [4] |
| Fine branch fraction | RamdaB | - | 0.19 | [4] |
| Min height-diameter | HdMin | - | 80 | [2] |
| Max height-diameter | HdMax | - | 120 | [2] |
| Max foliage | MaxGam |  | 0.03 | E |
| Age Max | AgeMax | years | 120 | E |
| Root loss ratio | GamaR | - | 0.0054 | D |
| Normal mortality ratio | MiuNorm | - | 0.005 | D |
| Crowding mortality ratio | MiuCrowd | - | 0.01 | D |
| Stem loss ratio | GamaS | - | 0 | D |

* Sources: D = Default; O = Observed.

1. Zhang, J., Chu, Z., Ge, Y., Zhou, X., Jiang, H., Chang, J., Peng, C., Zheng, J., Jiang, B., Zhu, J., 2008. TRIPLEX model testing and application for predicting forest growth and biomass production in the subtropical forest zone of China's Zhejiang Province. *Ecological Modelling*, 219, 264-275.
2. Zhou X.L., Peng C.H., Dang Q.L.,2004. Assessing the generality and accuracy of the TRIPLEX model using in situ data of boreal forests in central Canada. *Environmental Modelling and Software*, 19(1): 35-46.
3. Jiao, X.M., Xiang, W.H., Tian, D.L., 2005. Carbon storage of forest vegetation and its geographical distribution in Hunan Province. *Journal of Central South Forestry University*, 1, 4-8.
4. Zhang J., Chu Z.Y., Ge Y., Zhou X.L., Jiang H., Chang J., Peng C.H., Zheng J.W., Jiang B., *M* Zhu J.R., Yu S.Q., 2008. TRIPLEX model testing and application for predicting forest growth and biomass production in the subtropical forest zone of China's Zhejiang Province. *Ecological odellin*g, 219(3): 294-275.
