# Supplementary material for: Stand carbon storage and net primary production in China’s subtropical secondary forests are predicted to increase by 2060
Source: Carbon Balance Manag. 2022 May 26;17:6. doi: 10.1186/s13021-022-00204-y (PMC9134694; doi:10.1186/s13021-022-00204-y)
Supplement: Supplementary file 6 — Additional file 6. Descriptive statistics for the spatial heterogeneity of stand growth by forests in all selected permanent forest plots in Hunan Province in 2014. Including stand-structure variables: stem density (stems ha-1), stand age (year), diameter at breast height (DBH, cm), height (m), NPP (t ha-1 yr-1) and stand biomass (t ha-1). [file 13021_2022_204_MOESM6_ESM.doc]

**Additional file F.** Descriptive statistics for the spatial heterogeneity of stand growth by forests in all selected permanent forest plots in Hunan Province in 2014. Including stand-structure variables: stem density (stems ha^-1^), stand age (year), diameter at breast height (DBH, cm), height (m), NPP (t ha^-1^ yr^-1^) and stand biomass (t ha^-1^).

| Variables | Forest types | Mean | Standard deviation | Minimum | Maximum |
| --- | --- | --- | --- | --- | --- |
| Stem density  (stems ha^-1^) | Evergreen broad-leaved forest | 1303.505 | 734.852 | 180 | 4258 |
|  | Deciduous broad-leaved forest | 1189.961 | 634.609 | 90 | 3478 |
|  | Deciduous and evergreen broad-leaved mixed forest | 1285.335 | 677.026 | 135 | 3553 |
|  | Conifer and broad-leaved mixed forest | 1231.796 | 608.401 | 75 | 4078 |
|  | Total | 1248.291 | 648.662 | 75 | 4258 |
| Stand age  (year) | Evergreen broad-leaved forest | 25.581 | 17.901 | 5 | 84 |
|  | Deciduous broad-leaved forest | 17.735 | 9.869 | 5 | 48 |
|  | Deciduous and evergreen broad-leaved mixed forest | 19.760 | 13.224 | 5 | 82 |
|  | Conifer and broad-leaved mixed forest | 18.033 | 8.796 | 4 | 46 |
|  | Total | 19.310 | 11.885 | 4 | 84 |
| DBH (cm) | Evergreen broad-leaved forest | 12.615 | 4.841 | 1.5 | 27.3 |
|  | Deciduous broad-leaved forest | 11.384 | 3.676 | 6.5 | 30.5 |
|  | Deciduous and evergreen broad-leaved mixed forest | 11.089 | 4.079 | 5.5 | 32.2 |
|  | Conifer and broad-leaved mixed forest | 8.463 | 2.278 | 2.9 | 16.2 |
|  | Total | 10.226 | 3.796 | 1.5 | 32.2 |
| Height (m) | Evergreen broad-leaved forest | 8.717 | 2.978 | 2 | 16.8 |
|  | Deciduous broad-leaved forest | 8.492 | 2.517 | 2.2 | 16.7 |
|  | Deciduous and evergreen broad-leaved mixed forest | 8.249 | 2.464 | 2.5 | 21 |
|  | Conifer and broad-leaved mixed forest | 8.464 | 2.277 | 2.9 | 16.2 |
|  | Total | 8.430 | 2.459 | 2 | 21 |
| NPP  (t ha^-1^ yr^-1^) | Evergreen broad-leaved forest | 3.844 | 1.391 | 1.28 | 6.9 |
|  | Deciduous broad-leaved forest | 3.802 | 1.477 | 1.78 | 7.67 |
|  | Deciduous and evergreen broad-leaved mixed forest | 3.478 | 1.176 | 1.73 | 7.32 |
|  | Conifer and broad-leaved mixed forest | 2.799 | 1.064 | 1.62 | 7.34 |
|  | Total | 3.296 | 1.290 | 1.28 | 7.67 |
| All biomass  (t ha^-1^) | Evergreen broad-leaved forest | 114.228 | 97.221 | 0.579 | 397.338 |
|  | Deciduous broad-leaved forest | 86.089 | 74.372 | 0.82 | 408.68 |
|  | Deciduous and evergreen broad-leaved mixed forest | 68.315 | 67.120 | 0.32 | 477.57 |
|  | Conifer and broad-leaved mixed forest | 33.968 | 28.501 | 1.01 | 183.33 |
|  | Total | 61.939 | 66.071 | 0.32 | 477.57 |
