# Supplementary material for: Stand carbon storage and net primary production in China’s subtropical secondary forests are predicted to increase by 2060
Source: Carbon Balance Manag. 2022 May 26;17:6. doi: 10.1186/s13021-022-00204-y (PMC9134694; doi:10.1186/s13021-022-00204-y)
Supplement: Supplementary file 7 — Additional file 7. Descriptive statistics for spatial heterogeneity of climate and forest stand structure in all selected permanent forest sample plots in Hunan Province. Site variable: elevation (above mean sea level, m); climatic variables: annual average air temperature (°C), annual rainfall (mm yr-1) and the annual average vapor pressure deficit (mbar) between 2000 and 2014. [file 13021_2022_204_MOESM7_ESM.doc]

**Additional file G.** Descriptive statistics for spatial heterogeneity of climate and forest stand structure in all selected permanent forest sample plots in Hunan Province. Site variable: elevation (above mean sea level, m); climatic variables: annual average air temperature (°C), annual rainfall (mm yr^-1^) and the annual average vapor pressure deficit (mbar) between 2000 and 2014.

| Variables | Forest type | Mean | Standard deviation | Minimum | Maximum |
| --- | --- | --- | --- | --- | --- |
| Elevation (m) | Evergreen broad-leaved forest | 525.645 | 354.030 | 80 | 1440 |
|  | Deciduous broad-leaved forest | 551.445 | 278.468 | 80 | 1470 |
|  | Deciduous and evergreen broad-leaved mixed forest | 531.659 | 360.453 | 23 | 1740 |
|  | Conifer and broad-leaved mixed forest | 460.931 | 298.998 | 30 | 1560 |
|  | Total | 505.425 | 322.614 | 23 | 1740 |
| Yearly average  atmosphere  temperature (℃) | Evergreen broad-leaved forest | 15.928 | 1.543 | 10.263 | 18.170 |
|  | Deciduous broad-leaved forest | 15.139 | 1.371 | 10.807 | 18.503 |
|  | Deciduous and evergreen broad-leaved mixed forest | 15.508 | 1.664 | 9.477 | 18.234 |
|  | Conifer and broad-leaved mixed forest | 15.748 | 1.335 | 10.752 | 18.601 |
|  | Total | 15.586 | 1.489 | 9.477 | 18.601 |
| Annual  rainfall (mm) | Evergreen broad-leaved forest | 1543.968 | 196.842 | 1253.943 | 2029.211 |
|  | Deciduous broad-leaved forest | 1464.22 | 145.949 | 1196.995 | 2149.887 |
|  | Deciduous and evergreen broad-leaved mixed forest | 1506.202 | 193.692 | 1195.763 | 2128.925 |
|  | Conifer and broad-leaved mixed forest | 1463.345 | 174.218 | 1164.79 | 2249.266 |
|  | Total | 1485.122 | 180.244 | 1164.79 | 2249.266 |
| Yearly average vapour pressure deficit (mbar) | Evergreen broad-leaved forest | 3.601 | 0.417 | 2.437 | 4.332 |
|  | Deciduous broad-leaved forest | 3.422 | 0.333 | 2.486 | 4.270 |
|  | Deciduous and evergreen broad-leaved mixed forest | 3.526 | 0.433 | 2.486 | 4.304 |
|  | Conifer and broad-leaved mixed forest | 3.582 | 0.375 | 2.564 | 4.315 |
|  | Total | 3.538 | 0.396 | 2.437 | 4.332 |
