# Supplementary material for: Stand carbon storage and net primary production in China’s subtropical secondary forests are predicted to increase by 2060
Source: Carbon Balance Manag. 2022 May 26;17:6. doi: 10.1186/s13021-022-00204-y (PMC9134694; doi:10.1186/s13021-022-00204-y)
Supplement: Supplementary file 8 — Additional file 8. Simulation errors of TRIPLEX1.6 applied to subtropical forest ecosystems in southeastern China, comparing density (stems ha−1), DBH (cm), NPP (t ha-1 yr-1) and C storage (t C ha−1) between modeled values and forest inventory data collected from 875 forest stands. [file 13021_2022_204_MOESM8_ESM.doc]

**Additional file H. Simulation errors of TRIPLEX1.6 applied to subtropical forest ecosystems in southeastern China, comparing density (stems ha^−1^), DBH (cm), NPP (t ha^-1^ yr^-1^) and C storage (t C ha^−1^) between modeled values and forest inventory data collected from 875 forest stands.**

| Forest Types | n | P-Value | DBH (cm) | | Stem density  (stems ha^-1^) | | C storage  (t C ha^-1^) | | NPP (t ha^-1^ yr^-1^) | |
| --- | --- | --- | --- | --- | --- | --- | --- | --- | --- | --- |
|  |  |  | R^2^ | Se | R^2^ | Se | R^2^ | Se | R^2^ | Se |
| CBF | 360 | 0.001 | 0.961 | 0.13 | 0.998 | 31.80 | 0.871 | 1.64 | 0.986 | 0.05 |
| DBF | 155 | 0.001 | 0.978 | 0.25 | 0.991 | 50.06 | 0.920 | 4.14 | 0.958 | 0.14 |
| DEF | 267 | 0.001 | 0.975 | 0.23 | 0.998 | 41.08 | 0.910 | 4.01 | 0.953 | 0.07 |
| EBF | 93 | 0.001 | 0.970 | 0.40 | 0.993 | 69.48 | 0.915 | 8.01 | 0.905 | 0.20 |

Note: n, number of stands; *R*^2^, coefficient of determination; Se, standard error of the predicted value for each observed value in their regression, which is a measure of the amount of error in the prediction for an individual observation.
