# Supplementary material for: Stand carbon storage and net primary production in China’s subtropical secondary forests are predicted to increase by 2060
Source: Carbon Balance Manag. 2022 May 26;17:6. doi: 10.1186/s13021-022-00204-y (PMC9134694; doi:10.1186/s13021-022-00204-y)

**Additional file I.** Variation of monthly average temperature and precipitation in Hunan Province from 2000 to 2014.


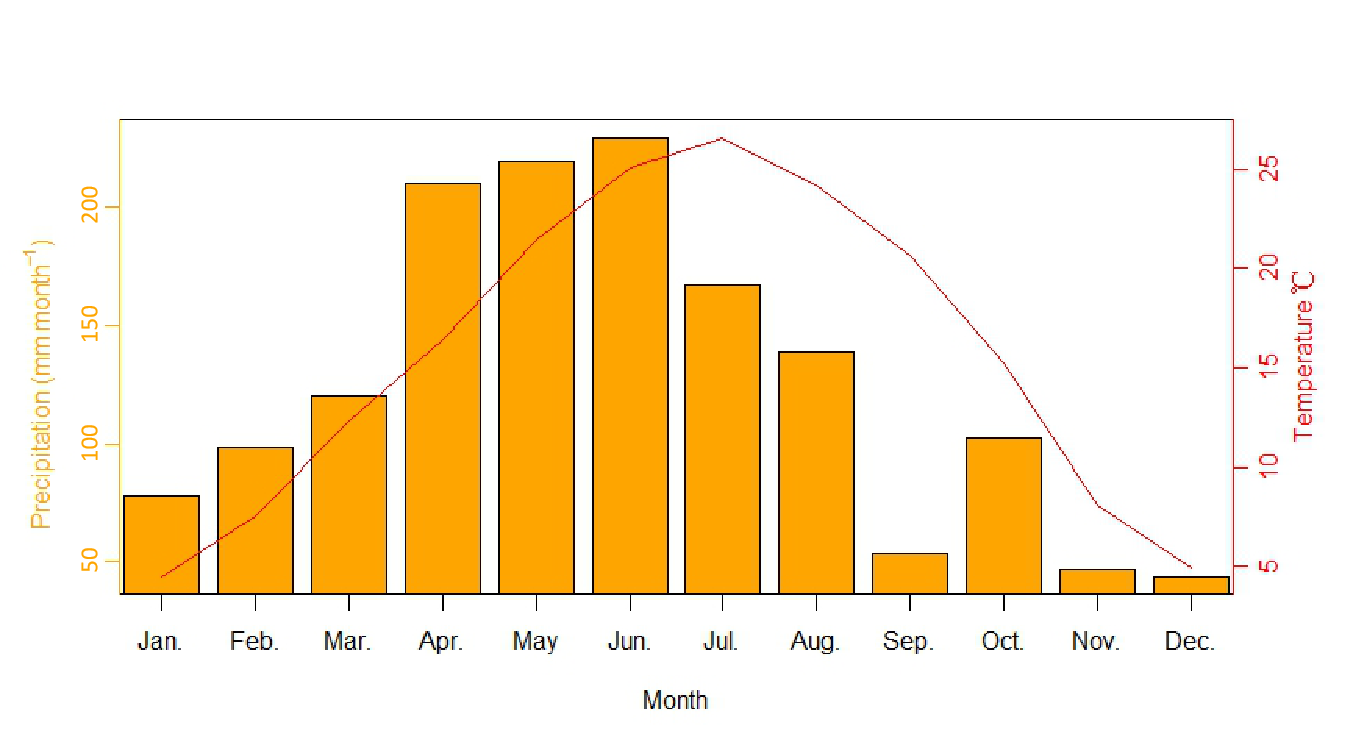

Supplement: Supplementary file 9 — Additional file 9. Variation of monthly average temperature and precipitation in Hunan Province from 2000 to 2014. [file 13021_2022_204_MOESM9_ESM.doc]
