# Supplementary material for: Stand carbon storage and net primary production in China’s subtropical secondary forests are predicted to increase by 2060
Source: Carbon Balance Manag. 2022 May 26;17:6. doi: 10.1186/s13021-022-00204-y (PMC9134694; doi:10.1186/s13021-022-00204-y)
Supplement: Supplementary file 10 — Additional file 10. Variation of monthly average temperature and precipitation in Hunan Province from 2000 to 2014. [file 13021_2022_204_MOESM10_ESM.doc]

**Additional file J.** Comparison of C storage density in 2014 for subtropical secondary forests of the present study with the data in the publish [literature](../../../../D:/Program%20Files%20(x86)/Youdao/Dict/8.9.9.0/resultui/html/index.html#/javascript:;)s.

| Forest type | C storage (t C ha^−1^) | Site | Reference | Source |
| --- | --- | --- | --- | --- |
| Evergreen broad-leaved forest | 55.49 | Hunan | This study | Inventory |
|  | 126.4 | Wuling mountain, Hunan | Xu et al., 2015 |  |
|  | 41.72 | Zhejiang | Xu et al., 2018 |  |
|  | 44.59 | Zhejiang | Zhang et al., 2007 |  |
|  | 66.5 | China | Fang et al., 1996 |  |
| Deciduous and evergreen broad-leaved mixed forest | 41.09 | Hunan | This study |  |
| Deciduous broad-leaved forest | 40.36 | Hunan | This study |  |
|  | 20.28 | Zhejiang | Xu et al., 2018 |  |
|  | 38.13 | China | Fang et al., 1996 |  |
| Coniferous and broad-leaved mixed forest | 28.14 | Hunan | This study |  |
|  | 35.03 | Zhejiang | Zhang et al., 2007 |  |
|  | 48.80 | China | Fang et al., 1996 |  |
| Average | 37.17 | Hunan | This study |  |
|  | 25.00 | Hunan | Chen et al., 2019 |  |
|  | 29.70 | Central China | Zhao et al., 2019 |  |
